# Going Green Is Exhausting for Dark Personalities but Beneficial for the Light Ones: An Experience Sampling Study That Examines the Subjectivity of Pro-environmental Behavior

**DOI:** 10.3389/fpsyg.2022.883704

**Published:** 2022-04-14

**Authors:** Jana Sophie Kesenheimer, Tobias Greitemeyer

**Affiliations:** Institute for Psychology, University of Innsbruck, Innsbruck, Austria

**Keywords:** pro-environmental behavior, costs and benefits estimates, dark tetrad, light triad, personality and behavior

## Abstract

Study 1 examined how personality and attitudes are related to daily pro-environmental behavior (PEB) and whether these relationships are moderated by perceived behavioral costs and benefits. One hundred and seventy-eight participants responded to scales measuring the dark and light side of personality, as well as their pro-environmental attitude. Afterward, they were notified three times a day for 7 days in a row. Each time they reported their PEB that had occurred in the past four hours and indicated their behavioral costs and benefits. Multilevel analyses showed a positive relationship between the frequency of PEB and the light triad of personality and pro-environmental attitude, while the dark tetrad was negatively related to PEB. Unexpectedly, less environmentally aware participants reported to engage in PEB with higher costs and lower benefits than did pro-environmental participants. A second study (*N* = 159) suggests that less environmentally aware people do not actually engage in PEB with high costs and low benefits, but rather that they only perceive their behavior to be costly and of little benefit. Overall, our findings suggest that the way people perceive their daily PEB is not necessarily shared by others.

## Introduction

According to the latest climate report, the sixth assessment cycle of the Intergovernmental Panel on Climate Change (IPCC, 2022), it is very likely that the temperature will rise by more than 1.5 degrees Celsius until 2030. This would mean that the European climate targets would not be met. Climate change is on the rise and can only be stopped by changing human behavior. Pro-environmental behavior (PEB) defines any behavior that has a positive impact on the natural environment, such as recycling, buying seasonal and regional food, a vegetarian or vegan diet, and reducing water and energy consumption (Steg and Vlek, 2009). These and similar household behaviors can save about 7% of national carbon emissions annually, which means, for example, 123 million tons less carbon dioxide in the United States (Dietz et al., 2009). Therefore, personal PEB is crucial to prevent further effects of the climate crisis. However, although climate change is one of the greatest threats to humanity, it is often difficult for people to adapt to “green” behavior in their everyday life (Kollmuss and Agyeman, 2002).

Previous research identified different types of PEB, for example, distinguishing between conservation lifestyle behaviors, social environmental protection, environmental citizenship, and land stewardship (Larson et al., 2015), each of which can take place more or less in a private or public setting (Lange and Dewitte, 2019). Most of the measures in the present study, researched by an ecological momentary assessment throughout the day, pertain to conservation behaviors that occur within the private sphere. For example, people mostly reflected on food and nutrition, energy and water conservation, travel modes, and garbage and plastic avoidance in their research diaries. These behaviors can be crucial in shifting to a lifestyle leading to reduced carbon emissions. For example, switching to a sustainable diet can save 500 kilograms of CO_2_ per year for one person (Cologna et al., 2022). Therefore, it is imperative for psychological research to understand personal and contextual variables that shape PEB (Steg and Vlek, 2009), in order to support the goal of sustainable living.

In Study 1, we examined how the pro-environmental attitude and dark and light personalities of people are related to these PEBs in daily life. We hypothesized that a pro-environmental attitude and light personality (and low scores on the dark personality) would characterize “pro-environmental people” who engage in PEB in daily life, while those with low scores on pro-environmental attitudes and light personalities and high scores on dark personalities would behave less pro-environmentally. Another goal was to test how the perceived costs and benefits affect people’s PEB. We anticipated that pro-environmental people would behave pro-environmentally even with high associated costs and low benefits. In contrast, less environmentally aware people would only behave environmentally friendly if the costs are low and/or benefits are high. To put it differently, when the costs are low and/or the benefits are high, a person’s attitude and personality do not predict whether the person engages in PEB (e.g., Wyss et al., 2022). In contrast, when costs are high and/or benefits are low, only pro-environmental people engage in PEB. As will be seen, whereas we did find the former (i.e., how pro-environmental and less pro-environmentally aware people differ in their daily life PEB), the moderation effects were not supported by the data.

Study 2 therefore examined how actors and observers differ in the perception of the actor’s PEB as a function of the actor’s pro-environmental attitude and personality. We reasoned and found that less pro-environmentally aware people perceive their PEB to be more costly and less beneficial than pro-environmental people and that this difference is mainly due to different perceptions rather than how PEB actually differs in terms of costs and benefits. Overall, our research sheds some light on who is more likely to behave pro-environmentally and on how subjective the perception of the costs and benefits of PEB is.

### The Role of Personality and Attitude in Shaping PEB

Environmental attitude focuses on general cognitive and affective components of environmental protection (Bamberg, 2003). In general, the assumption that attitudes shape actual behavior is widely recognized in psychological research (Bechler et al., 2021). Nevertheless, the exact circumstances under which attitudes shape behavior, and to what extent, are still being researched and the relationship between attitude and behavior appears to be complex (Bechler et al., 2021). For example, the “attitude–behavior gap” (Kollmuss and Agyeman, 2002, p. 246) suggests that concern about environmental problems is not necessarily reflected in behavior. Still, it has been shown that attitude plays a predominant role in predicting PEB (Kesenheimer and Greitemeyer, 2021a).

From a person-centered perspective and in addition to pro-environmental attitudes, there are several determinants of PEB. For example, basic personality traits, in particular honesty–humility and openness to experience go hand in hand with PEB (e.g., Brick and Lewis, 2016; Soutter et al., 2020; Soutter and Mõttus, 2021; Kesenheimer and Greitemeyer, 2021a). Honesty–Humility is characterized by being sincere, fair, and humble (Soutter et al., 2020). High levels of Openness characterize intellectual, imaginative, and independent-minded persons (Soutter et al., 2020). Both traits were found to be beneficial in environmentally friendly decision-making. However, beyond this broad view of personality, there is a lack of knowledge about the more extreme spectra. Concerning the “dark” and the “light” sides of personality, research about their relations to PEB is scarce (Naderi and Strutton, 2015; Kesenheimer and Greitemeyer, 2021b).

The dark tetrad of personality consists of Machiavellianism, narcissism, psychopathy, and sadism, and relates to self- and other aggression, antisocial behavior, and suicidal tendencies (Chabrol et al., 2015; Međedović and Petrović, 2015; Greitemeyer and Sagioglou, 2021). Compared to the basic big five taxonomy of personality, “dark personalities were associated with low honesty–humility and agreeableness” (Book et al., 2016; p. 271). More specifically, the definition of Machiavellianism is based on the 16th-century philosopher Nicolo Machiavelli and describes people who are “cynical, unprincipled, believe in interpersonal manipulation as the key for life success, and behave accordingly” (Furnham et al., 2013, p. 201). Recent results showed a negative association of Machiavellianism and psychopathy with environmental attitudes (Huang et al., 2019). In addition, Machiavellianism predicted the justification of eating meat (Mertens et al., 2021). Psychopathy describes people who are exceptionally impulsive, looking for thrills and have very little empathy (Lilienfeld and Andrews, 1996; Furnham et al., 2013). Narcissism is characterized by self-enhancement and status seeking, and reduces daily PEB if “green” behavior has been shown for predominantly altruistic reasons (Kesenheimer and Greitemeyer, 2021b). On the other hand, it has also been shown that narcissism can increase PEB in certain situations when PEB goes along with enhanced status or other egoistic benefits (Griskevicius et al., 2010; Kesenheimer and Greitemeyer, 2021b). Sadism is the fourth dimension of the dark tetrad (Chabrol et al., 2015; Međedović and Petrović, 2015). People who score relatively high on everyday sadism take pleasure in seeing others suffer harm (e.g., Greitemeyer et al., 2019). It has been discussed (but not yet examined) that everyday sadism is negatively associated with attitudes toward the environment (Mertens et al., 2021). Taken together, the dark tetrad and PEB should be negatively related due to this socially aversive nature (Paulhus and Williams, 2002; Huang et al., 2019).

Located on the opposite spectrum of personality, the light triad is made up of faith in humanity, humanism, and Kantianism (Kaufman et al., 2019). These traits are defined as a tendency to treat “people as ends unto themselves,” to appreciate “the dignity and worth of each individual” and the belief that people are fundamentally good (Kaufman et al., 2019, p. 1). This light triad has been described as a more beneficial, positive, and growth-oriented side of the personality when compared to its counterpart, the dark tetrad. Rather, it reflects the basic assumptions of positive psychology (Lukić and Živanović, 2021). Compared to the big five personality traits (Goldberg, 1990), previous research showed weak positive correlations of the light triad and openness, conscientiousness, and extraversion, but a slightly negative correlation to neuroticism, while there was a strong positive association with agreeableness (Gerymski and Krok, 2019; Kaufman et al., 2019; Lukić and Živanović, 2021). Despite these relationships, as well as its strong positive correlation to the honesty–humility factor of the Hexaco model (Ashton and Lee, 2007), it was shown that the light triad is distinctive of these constructs (Kaufman et al., 2019).

The light sight of personality has not yet been explored in relation to daily PEB, but there is evidence for a positive correlation: “green” entrepreneurship was discussed to be related to the light triad to explain the environmentally aware economy of companies like Veja and Vaude (Cooke, 2020). In addition, the light personality construct showed negative correlations with self-enhancement and strong positive correlations with honesty–humility (Kaufman et al., 2019), which is crucial since PEB is often characterized by overcoming self-sacrifice (Prinzing, 2020). On top, both, honesty–humility and openness, as described earlier, positively correlate to the light triad (Kaufman et al., 2019). These previous research findings lead to the assumption that the light triad might indeed be beneficial for PEB. The goal of the present research was to examine the extreme spectra of subclinical levels of personality by examining the relationship of the dark tetrad and light triad to PEB in daily life. In this way, evidence can be found for personal and contextual factors in general that influence decisions about an environmentally conscious lifestyle.

### The Moderating Impact of Perceived Costs and Benefits

Besides a person-centered point of view, rational choice models, such as the theory of planned behavior (Ajzen, 1991) and theories of public goods, assume that costs and benefits are decisive context factors of PEB. These models typically focus on the assumption that people act to maximize individual benefit and minimize personal costs, in line with the “homo oeconomicus” model (Turaga et al., 2010). Costs and benefits were also discussed to explain the attitude–behavior gap in the past (Fietkau and Kessel, 1981; Kollmuss and Agyeman, 2002). For example, recycling is easy when suitable recycling facilities are nearby, but the same behavior is much more difficult when recycling facilities are not available and therefore require a high level of pro-environmental awareness. As another example, some green behaviors have personal benefits (e.g., saving money by drinking tap water), while others require more self-sacrifice (e.g., not consuming animal products). Previous results based on the theory of planned behavior show that perceived costs and benefits shape PEB in various situations, for example, when deciding on travel modes (Bamberg and Schmidt, 2003) and when recycling or composting household garbage (Taylor and Todd, 1995).

“Homo oeconomicus” (Turaga et al., 2010) would make very predictable decisions when it comes to environmentally aware decisions. If the personal costs associated with a particular behavior were greater than its benefits, homo oeconomicus would not exhibit this behavior. However, human behavior does not only depend on objective contextual factors. Contextual factors and personal determinants can interact so that a given context can have a moderating effect through the influence of attitudes, affects, or personal norms (Steg and Vlek, 2009). Pro-environmental attitude was found to be less predictive in high-cost situations (e.g., Wyss et al., 2022). If, for example, appropriate recycling facilities are available, this might only promote recycling behavior of already environmentally aware people. Previous research has even shown that “subjective enablers and constraints” depend on personality traits (specifically locus of control; Cleveland et al., 2020). Following these results, consumers with higher levels of internal environmental locus of control were more likely to perceive high enabling factors of PEB.

The reasoning leads to the assumption that perceived costs and benefits of PEB interact with personality and attitude. This means that the presumed direct influence of the dark and light side of personality as well as the influence of a pro-environmental attitude on PEB should be more pronounced in high- (compared to low-) cost situations and low- (compared to high-) benefit situations. In contextual circumstances with high costs and/or little benefit, the hurdle to behave in an environmentally aware manner is high. A strong pro-environmental attitude and a high level of light personality can then be particularly beneficial for PEB.

## The Present Investigation

Given the presumed interplay of contextual and personal determinants of PEB, which has also been suggested in previous research (Cleveland and Kalamas, 2015), it seemed important to examine them simultaneously. As far as we know, the interplay of dark and light personality, environmental attitudes, and cost and benefit aspects have not yet been subject within a single environmental psychological study. Previously outlined results on personal determinants of PEB were based on single-point self-report measurements of PEB and/or did not consider contextual aspects. It is known that such single-point self-reports are often distorted by memory errors and social desirability (Kollmuss and Agyeman, 2002; Lange and Dewitte, 2019). These deficiencies can be remedied with the ecological momentary assessment (EMA). EMA means repeatedly measuring people’s behavior in everyday life. This allows examining different contexts and points in time in order to improve the ecological validity. In addition, the recall bias is minimized, as the behavior is asked about in very short periods of time (Shiffman et al., 2008).

In a first study, participants reported the PEBs that occurred during the previous 4 h three times a day (at 12.00 pm, 4 pm, and 8 pm) for seven consecutive days. For each PEB mentioned, a personal assessment of situational costs and benefits was given. The participants also reported about their personality and attitudes.

**Figure 1:**
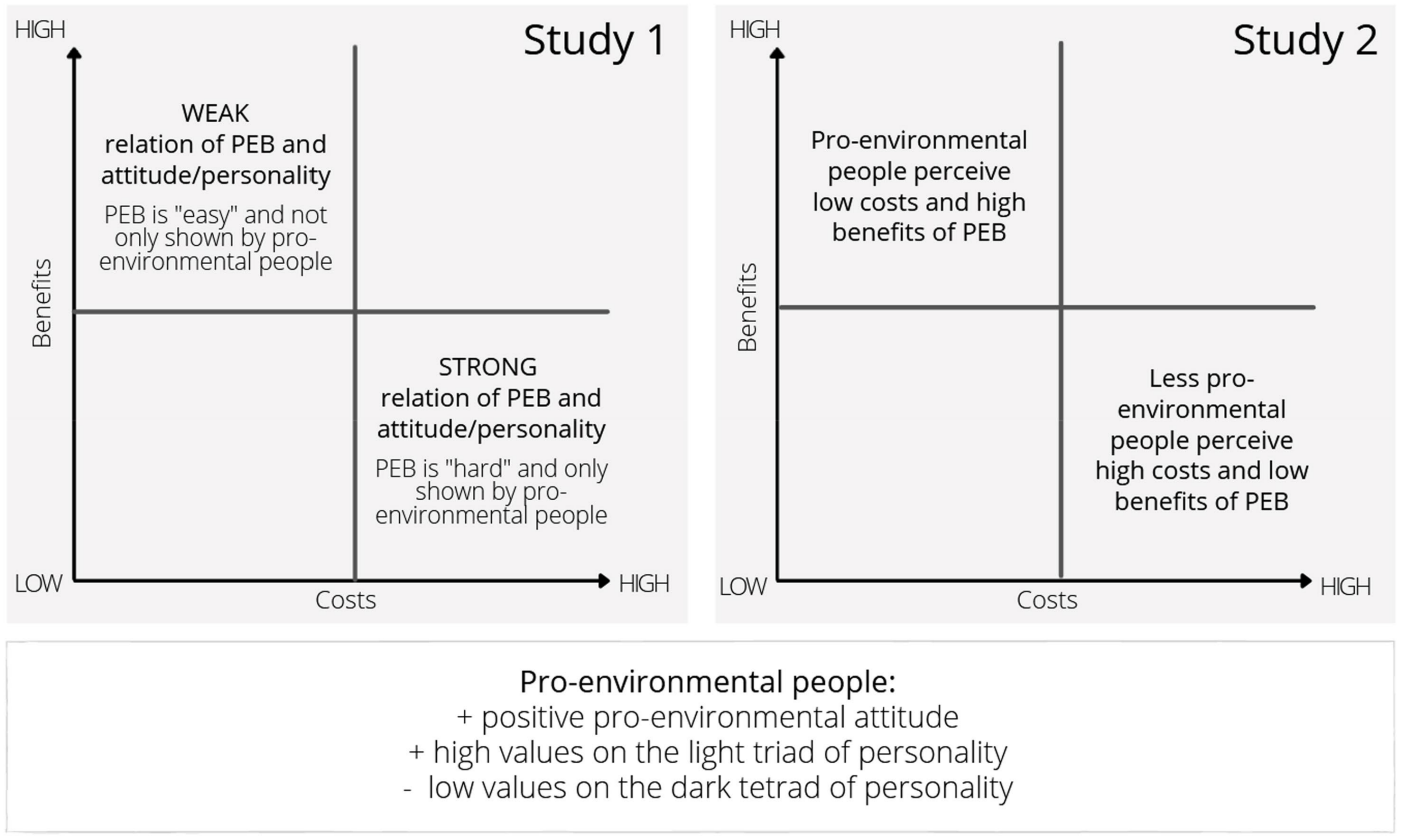
Summary of the studies’ assumptions.

Study 2 was then carried out to examine the subjectivity of situational cost and benefit estimates. We hypothesized that the perception of costs and benefits by pro-environmental and less pro-environmentally aware people would differ in that the former perceive lower costs and higher benefits than the latter. By comparing the ratings of actors and observers, we further investigated whether less pro-environmentally aware people are merely claiming that their PEB involves high costs and low benefits, or whether there is the broader pattern that less pro-environmentally aware people perceive PEB to be rather costly and of little benefit for the actor (even if they are not the actors themselves). The assumptions of the present investigation are summarized in Figure 1.

## Study 1

In Study 1, we hypothesized that pro-environmental attitude and the light triad are positively related to PEB, while the dark tetrad is negatively related. In addition, it was hypothesized that behavior costs and benefits would moderate these relations. In detail, we pre-registered^1^ the following hypotheses: it was expected that pro-environmental attitude is positively related to PEB (I). Furthermore, behavioral costs and benefits were expected to influence this relationship: the relationship between pro-environmental attitude and PEB was expected to be more pronounced in high-cost compared to low-cost situations (II) and in low-benefit compared to high-benefit situations (III), respectively. Concerning personality, it was assumed that the dark tetrad is negatively related to pro-environmental attitude (IVa) and PEB (IVb), whereas the light triad should be positively related to pro-environmental attitude (Va) and PEB (Vb). Here, too, it was expected that behavior costs and benefits moderate these relationships: the relationship between the dark tetrad and PEB should be more pronounced in high-cost compared to low-cost situations (VI), and accordingly more pronounced in low- compared to high-benefit situations (VII). Similarly, the relationship between the light triad and PEB should be more pronounced in high-cost vs. low-cost situations (VIII), and more pronounced in low-benefit vs. high-benefit situations (IX).

### Method

#### Participants

We advertised this EMS study in a university newsletter for students and employees, in university courses, *via* social media (Facebook), and in our personal environment. One hundred and seventy-eight participants took part in this study. Since two participants did not answer at least one of three items correctly in the attention tests, they were excluded from the data analysis. The remaining sample (*N* = 176) consisted of 115 women, 47 men, and 14 people with diverse gender or no answer on gender. Their mean age was 23.87 years (*SD* = 6.59) and ranged from 18 to 68 years. The sample can be described as academic, as 126 participants stated a high school degree, and 30 participants stated a university degree as their highest educational qualification. In addition, the average political orientation was 2.78 (*SD* = 1.60), indicated on a Likert scale from 0 to 10, with lower numbers indicating a more left-wing political orientation. Most of the participants (*N* = 131) earned less than 1,000€ a month.

#### Procedure

The entire study was carried out with an app (SEMA3; Koval et al., 2019) that notified the participants on their personal smartphone. After the initial survey, participants were notified at 12.00 noon, 4 pm, and 8 pm for seven consecutive days. Thus, they were able to answer on 21 short surveys in total about their PEB and its related costs and benefits. By finishing the study, the participants could receive course credit or take part in a raffle to win one of three cash prizes of 50 Euros as a reward.

#### Measures

At the initial survey right after registration, the participants were asked to answer on surveys about the light triad (12 items; German translation of Kaufman et al., 2019; Kantianism, faith in humanity, humanism) and the short dark tetrad of personality (28 items; Paulhus et al., 2020; sadism, narcissism, Machiavellianism, and subclinical psychopathy). Example items are from the light triad scale: “I prefer honesty over charm” (Kantianism), “I tend to see the best in people” (faith in humanity), and “I tend to treat others as valuable” (humanism). From the dark tetrad, example items are “I hurt others for my own pleasure” (sadism), “People see me as a natural leader” (narcissism), “I love it when a tricky plan succeeds” (Machiavellianism), and “People who mess with me always regret it” (psychopathy). In addition, pro-environmental attitude was assessed using the German adaption of the New Environmental Paradigm Scale (15 items; German version by Schleyer-Lindenmann et al., 2018; developed by Dunlap et al., 2000). An exemplary item is “People are badly abusing the environment.” The answers were given uniformly on a six-point scale (“*I do not agree at all*”—0 to “*I fully agree*”—5). Additionally, socio-demographic data (age, gender, education, political orientation, and financial net income) were collected. These covariates have proven to be influential in previous studies on PEB (Kesenheimer and Greitemeyer, 2021a,b).

During the daily notifications, participants were asked if they had “acted pro-environmentally at least once in the past 4 h.” As a reminder, it was repeated to “think of the areas of consumption and nutrition, transport and services, use of resources and recycling or waste disposal.” They were then able to name up to seven specific PEBs that occurred (e.g., choosing staircase over an elevator for environmental reasons). In addition, they rated costs and benefits associated with these behaviors on a 0- to 10-point scale (“For the behavior you just mentioned, your behavioral costs [benefits] were…”; “*very low*—0 to *very high*—10”). Before that, they were given a brief explanation of what behavior costs and benefits are. “Pro-environmental behavior can be complex (e.g., strenuous, time-consuming, expensive, and poor taste), which is described as behavioral costs. On the other hand, pro-environmental behaviors can also have a personal benefit (e.g., save money, give a good picture of yourself, and you feel better).”

### Results

The open-source software R, version 3.6.2 (R Core Team, 2018), was used to analyze the data. In the period from March 3 to April 14, 2021, 4,019 PEBs were collected in 1653 diary entries, meaning that each participant reported an average of 9.39 momentary assessments, each containing around 2.54 behaviors (arithmetic means). In detail, 32% of mentioned PEBs related to food intake (e.g., vegetarian or vegan diet, regional grown, and biological food), 22% related to energy and water conservation (e.g., taking a short shower instead of a bath and dress warmer instead of heating), 21% related to travel modes (e.g., public transport and taking the bike), and roughly 19% related to garbage and plastic (e.g., recycling and avoiding single-use plastic). Six percent of the mentions related to different behaviors (e.g., avoiding to buy new clothes, donate, and advice others). Thus, the behaviors mentioned were almost balanced in their areas of environmental impact.

#### Zero-Order Analyses

In a first step, scale reliabilities were tested with Cronbach’s alpha and normal distributions with Shapiro Wilk tests, which indicated a normal distribution for the dark tetrad (DT; *p* = 0.352), pro-environmental attitude (*p* = 0.225), Machiavellianism (*p* = 0.181) and narcissism (*p* = 0.098). Since the other variables were not normally distributed with *p* < 0.001, Spearman Rho correlations were performed consistently. Table 1 shows these correlations, means, and SDs. The diagonal line (grey) describes Cronbach’s alpha. Reliability of the light triad scale as a whole was *α* = 0.69 and *α* = 0.87 for the dark tetrad scale.

**Table 1:** Spearman’s rank rho correlations matrix as well as reliabilities (Cronbach’s alpha, diagonal) of Study 1 (*N* = 176).

		*M*	*SD*	1	2	3	4	5	6	7	8
1	PEA	3.66	0.51	0.74							
2	MACH	2.05	0.66	−0.26^**^	0.61						
3	NARC	2.2	0.75	−0.09^**^	0.25^**^	0.79					
4	SAD	0.95	0.7	−0.31^**^	0.51^**^	0.30^**^	0.71				
5	PSYC	1.46	0.78	−0.26^**^	0.57^**^	0.53^**^	0.56^**^	0.72			
6	HUMAN	3.72	0.66	0.03	−0.12^**^	−0.04^*^	−0.27^**^	−0.12^**^	0.6		
7	FIH	3.08	0.78	0.11^**^	−0.30^**^	−0.03	−0.24^**^	−0.21^**^	0.24^**^	0.68	
8	KANT	3.63	0.72	0.17^**^	−0.44^**^	−0.11^**^	−0.26^**^	−0.32^**^	0.24^**^	0.20^**^	0.52

M, arithmetic mean; SD, standard deviation; PEA, pro-environmental attitude; MACH, Machiavellianism; NARC, narcissism; SAD, sadism; PSY, psychopathy; HUMAN, humanism; FIH, faith in humanity; and KANT, Kantianism.

* Level of significance: *p* < 0.05.

** Level of significance: *p* < 0.01.

#### Relations With PEB

Due to the hierarchical structure of the data, consisting of up to 21 entries per person (7 days x three measures per day), multilevel models were carried out for the statistical analyses with repeated measurements. We used the R packages nlme (Pinheiro et al., 2021) and lme4 (Bates et al., 2015) for these multilevel analyses. In all multilevel models, individual starting points (*y*-axis section indicating individual levels of the PEBs’ frequency) were assumed for better model adaption (see the Bayesian Information Criteria, *BIC*, for each model reported in the following). For linear regression models containing only one level, we report the F-statistics to indicate the predictive power of all independent variables in each model.

**Figure 2:**
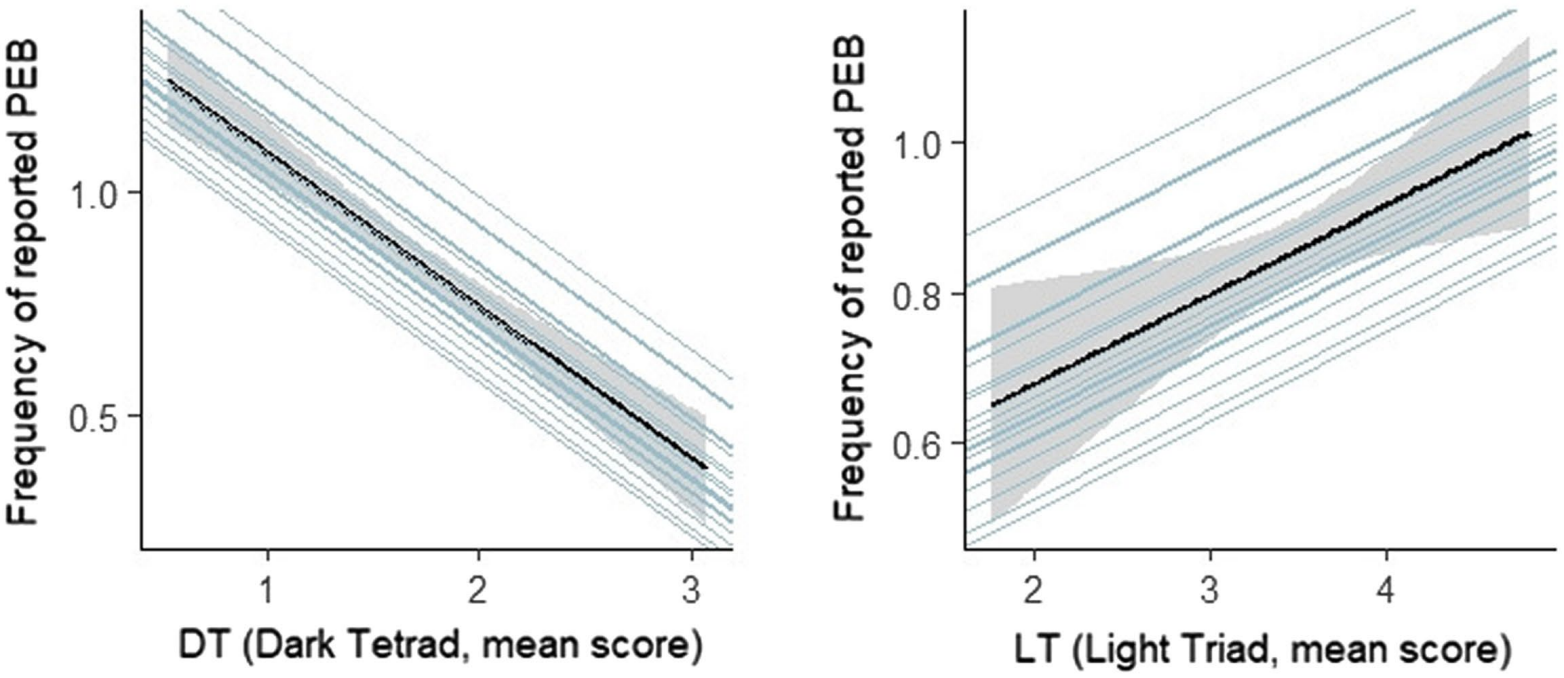
Multilevel models showing the negative relation of the dark tetrad (left) and the light triad’s positive relation (right) with the daily frequency of pro-environmental behavior (PEB). The shaded area displays the *CI*_95%_. The light-blue single lines display individual gradients.

Hypothesis 1 received support from the data: pro-environmental attitude positively correlated with PEB, *b* = 0.09 *CI*_95%_ [0.01, 0.18], *t*(3195) = 2.07, *p* = 0.038, *BIC* (Bayesian Information Criteria) = 10943.67. We also expected the dark tetrad to be negatively related to PEB (hypothesis 4). Indeed, the data supported this assumption, *b* = −0.33 *CI*_95%_ [−0.40, −0.24], *t*(3258) = −7.84, *p* < 0.001, *BIC* = 1109.75. In addition, we expected a positive relationship between the light triad and PEB (hypothesis 5). Data analysis supported this assumption, *b* = 0.10 *CI*_95%_ [0.01, 0.19], *t*(3343) = 2.23, *p* = 0.026, *BIC* = 11325.54. Figure 2 illustrates these relationships.

#### Relations With Pro-environmental Attitude

In line with the hypotheses 4a and 5a, a pro-environmental attitude was negatively associated with the dark tetrad, *b* = −0.34 *CI*_95%_ [−0.38, −0.31], *t*(3215) = −19.09, *p <* 0.001, and positively associated with the light triad, *b* = 0.09 *CI*_95%_ [0.05, 0.13], *t*(3215) = 5.33, *p* < 0.001.

#### The Role of Behavior Cost and Benefits

Further, we expected the costs and benefits of PEB to influence the relationships of attitude, the dark and the light side of personality with PEB. We investigated the relation of behavior costs and benefits with personality and attitude traits using multilevel models. Taking into account the frequency of mentioned PEBs, we added it as a covariate. Since frequency was linked to cost and benefit estimates that were only given for mentioned PEBs (and not for non-mentions), it was not possible to use behavioral frequency as a dependent variable in regression models. Reporting higher costs (benefits) was therefore also associated with a higher frequency of costly (beneficial) PEB. We therefore tested the hypotheses 2 and 3 and the hypotheses 6–9 by examining the relationships between cost and benefit estimates and personality and attitude traits. The frequency was a significant covariate in all analyses.

We reasoned that the dark tetrad should be associated negatively with reported behavioral costs and positively with reported behavioral benefits. If costs are low and/or benefits are high, then all participants should be inclined to engage in PEB. In the case of high costs and/or low benefits, on the other hand, only participants with relatively low values in the dark tetrad (high values in the light triad and/or pro-environmental attitude) should undertake engaging in PEB. Based on the same argument, we expected that pro-environmental attitude and the light triad of personality are positively associated with costs and negatively with benefits.

First, the relation of behavior costs and pro-environmental attitude was investigated (hypothesis 2). Results of a multilevel model showed no relation of costs and pro-environmental attitude, *b* = 0.05 *CI*_95%_ [−0.15, 0.25], *t*(1367) = 0.50, *p* = 0.617, *BIC* = 5706.32. Thus, the data did not support this assumption. In addition, we tested whether pro-environmental attitude was negatively related to perceived behavioral benefits (hypothesis 3). Unexpectedly, we found a positive relationship, *b* = 0.46 *CI*_95%_ [0.16, 0.46], *t*(1367) = 3.05, *p* = 0.002, *BIC* = 6836.72.

To test whether behavioral costs and benefits moderate the relationship between the dark tetrad and PEB, we examined how the dark tetrad relates to perceived costs and benefits (hypotheses 6 and 7). We expected the dark tetrad to be associated negatively with costs and positively with benefits. However, multilevel models showed that participants with high values of the dark tetrad reported higher behavioral costs, *b* = 0.37 *CI*_95%_ [0.19, 0.55], *t*(1399) = 3.98, *p* < 0.001, *BIC* = 5832.21, and fewer behavioral benefits, *b* = −0.71 *CI*_95%_ [−0.98, −0.43], *t*(1399) = −5.08, *p* < 0.001, *BIC* = 6974.46.

In terms of the light triad, we expected a positive relationship to costs and a negative relationship to benefits. Here, too, both hypotheses were refuted by the data. When testing hypothesis 8, the light triad of personality was shown to correlate negatively with perceived costs, *b* = −0.62 *CI*_95%_ [−0.84, −0.40], *t*(1408) = −5.62, *p* < 0.001, *BIC* = 5842.32, and positively with perceived benefits, *b* = 1.19, *CI*_95%_ [0.87, 1.52], *t*(1408) = 7.22, *p* < 0.001, *BIC* = 7003.54.

### Discussion

On the one hand, Study 1 supported the hypotheses 1, 4a/b, and 5 a/b: the frequency of reported PEB in daily life was positively related to pro-environmental attitude and the light side of personality and negatively related to the dark tetrad. Accordingly, the pro-environmental attitude went hand in hand with the light triad but was negatively related to the dark tetrad. Thus, the data supported findings from previous literature on the negative relationship between the dark side of personality and PEB (Mertens et al., 2021; Kesenheimer and Greitemeyer, 2021b), as well as on a positive relationship between the light triad and PEB (Cooke, 2020).

On the other hand, the present results showed the exact opposite of what we had expected regarding the other hypotheses (2, 3, and 6–9). Perceived benefits were positively associated with pro-environmental attitude and the light triad scale, and negatively with the dark tetrad. Moreover, the perceived costs correlated positively with the dark tetrad and negatively with the light triad. Although these findings were statistically reliable and were based on a large number of observations, we doubt that people who are not environmentally aware actually behave more environmentally aware in costly situations with little personal benefit. Rather, as estimates of costs and benefits are subjective, we believe that some people (in particular, those who are not environmentally aware) do not accurately judge the extent to which their PEB entails (high) costs and (low) benefits. Hence, it is possible that the relationships of personality and attitude with “costly” or “beneficial” PEB were shown due to subjective perceptions. Thus, we anticipated that the result from Study 1, that non-environmentally aware participants were more likely to report PEB with high costs and little benefit than did pro-environmental participants, is due to the subjective assessment of costs and benefits and would not be confirmed in estimates by independent observers.

In Study 2, independent observers rated the costs and benefits of the PEBs reported by the participants (in the following, actors) in Study 1. This enabled us to investigate whether less environmentally aware people would estimate higher costs and lower benefits of PEB than pro-environmental people do. Such a finding would suggest that less environmentally aware people do not exhibit PEB with high costs and low benefits (as the findings of Study 1 could be interpreted), but rather, that the findings from Study 1 are mainly due to a high subjectivity of the perceived costs and benefits.

## Study 2

### Method

In the view of more than 4,000 entries in the EMS of Study 1, we summarized these behaviors in 102 categories (Table 2). Each participant in Study 2 rated all 102 categories concerning the estimated behavioral costs and benefits on the same 0- to 10-point scale as in Study 1 (0—*no costs at all* to 10—*very high costs*; 0—*no benefits at all* to 10—*very high benefits*). An introduction explained, “*behavioral costs are not just financial costs. For example, time resources may be required for pro-environmental behavior, or the behavior may require some effort or self-sacrifice. However, the same behavior can have benefits too: you can potentially save money, make a positive impression on others, feel better and add some social benefit*.” At the end of the survey, participants responded to the exact same variables as participants in Study 1: demographics, the light triad scale, the dark tetrad scale, and environmental attitude.

**Table 2:** Categories based on the behavior descriptions in Study 1 and their frequencies in Study 1, as well as average estimated behavior costs and benefits of Study 2.

Category	Absolut counts in Study 1	Percent of total counting (%)	Mean estimated costs (*SD*)	Mean estimated benefits (*SD*)
Vegetarian meal	385	15.35	4.83 (2.59)	7.87 (2.85)
Vegan meal	286	11.40	6.94 (2.74)	7.50 (3.10)
Travel by foot	165	6.58	5.64 (2.67)	7.82 (2.74)
Recycle	149	5.94	3.07 (2.16)	8.13 (3.06)
Travel by bike	146	5.82	4.79 (2.58)	8.49 (2.68)
Drink tap water	123	4.90	2.65 (2.16)	7.70 (3.01)
Avoid single-use plastic	116	4.63	3.90 (2.34)	8.08 (2.79)
Stairs (no elevator)	107	4.27	3.60 (2.34)	7.40 (2.97)
Public transportation	86	3.43	4.86 (2.41)	7.92 (2.81)
Shower (save water)	78	3.11	3.16 (2.35)	7.26 (3.30)
Quick shower	70	2.79	3.71 (2.21)	6.99 (2.93)
Regional foods	57	2.27	4.52 (2.53)	8.09 (2.61)
Bio foods	55	2.19	5.11 (2.41)	7.99 (2.43)
Turnoff light	52	2.07	2.08 (1.94)	7.79 (3.20)
Use leftovers (foods)	47	1.87	3.21 (2.14)	8.06 (2.77)
No plastic bag	40	1.59	2.12 (1.79)	7.96 (3.09)
No light (use daylight)	40	1.59	3.52 (2.46)	7.27 (3.15)
Glass instead of plastic package	24	0.96	3.92 (2.13)	7.73 (2.82)
No tumble dryer	21	0.84	3.94 (2.40)	7.45 (3.04)
Shower (no bath)	21	0.84	2.84 (2.21)	7.42 (2.98)
Plant milk (no cow milk)	19	0.76	4.12 (2.53)	6.84 (3.13)
Save electricity (saving mode)	18	0.72	2.86 (1.90)	7.06 (2.89)
Solid shampoo (no plastic)	18	0.72	4.62 (2.54)	6.56 (3.00)
Turn of heating (when leaving)	17	0.36	3.38 (2.51)	7.15 (3.13)
Stop heating during ventilation	16	0.64	3.10 (2.42)	6.90 (3.10)
No heating, dress warmer	15	0.60	5.15 (2.51)	6.59 (2.86)
Cold shower	15	0.60	5.86 (2.69)	5.88 (2.86)
2ndhand clothes	12	0.48	4.65 (2.44)	7.44 (2.79)
Cook (no convenience food)	12	0.48	5.20 (2.35)	7.43 (2.46)
2ndhand things	11	0.44	4.48 (2.26)	7.29 (2.77)
e-Car	11	0.44	6.57 (2.82)	7.02 (2.71)
No shopping	11	0.44	3.45 (2.38)	8.40 (2.80)
Recycle cigarette	10	0.40	2.66 (2.28)	7.60 (3.41)
Shared car ride	10	0.40	4.30 (2.37)	7.98 (2.70)
Turnoff unused electricity	10	0.40	2.70 (2.31)	7.68 (3.17)
Single wash (wash many clothes at a time)	9	0.36	2.85 (2.13)	7.71 (3.04)
Solid soap (no plastic)	9	0.36	3.45 (2.46)	6.40 (3.04)
Take the garbage others left behind	9	0.36	3.47 (2.58)	8.04 (2.94)
Eco mode washing machine	8	0.32	2.87 (2.07)	6.79 (2.92)
Hand wash (dishes)	8	0.32	5.62 (2.60)	5.00 (2.82)
No shower	8	0.32	7.55 (3.11)	4.58 (3.23)
Upcycling	8	0.32	5.34 (2.60)	7.42 (2.73)
Ecological cleaning products	7	0.28	4.04 (2.25)	6.99 (2.90)
Farmer (no supermarket)	7	0.28	5.55 (2.49)	7.89 (2.50)
Using Tupperware or similar	7	0.28	2.95 (2.01)	7.50 (2.97)
Compost	6	0.24	4.74 (2.83)	7.16 (2.73)
Read digital (no print)	6	0.24	3.90 (2.46)	6.99 (3.01)
Dumpster (passive)	6	0.24	4.74 (2.74)	7.01 (2.75)
Ecological care products (e.g., crème)	6	0.24	4.39 (2.34)	6.87 (2.89)
No hair dryer	6	0.24	3.09 (2.22)	6.87 (3.02)
Heating only one room	6	0.24	5.22 (2.51)	6.39 (2.71)
Plant vegetables	6	0.24	7.18 (2.63)	7.77 (2.52)
Bio meat	5	0.20	4.70 (2.56)	8.29 (2.51)
Ecological washing product	5	0.20	4.16 (2.50)	6.97 (2.87)
Advice or help others being pro-environmental	5	0.20	5.58 (2.80)	7.03 (3.03)
Recycle glass	5	0.20	3.09 (2.15)	7.49 (3.21)
Using renewable energy	5	0.20	7.01 (1.95)	7.58 (1.01)
Ignore best before date	4	0.16	3.77 (2.59)	7.81 (2.83)
Lid (to cook)	4	0.16	2.40 (1.76)	6.97 (2.96)
No lunch to go	4	0.16	4.16 (2.15)	7.83 (2.64)
Give away used things (present/donation)	4	0.16	3.11 (2.28)	7.91 (3.07)
Raw meal (instead of cooking)	4	0.16	5.78 (2.79)	5.27 (2.88)
Bee-wax instead of plastic package	3	0.12	4.70 (2.54)	6.60 (2.95)
Bottled beer (no cans)	3	0.12	2.86 (1.90)	6.94 (3.09)
DIY cosmetic products	3	0.12	6.74 (2.63)	5.84 (2.90)
Dumpster	3	0.12	6.04 (2.84)	6.76 (2.73)
Local shop (not online)	3	0.12	4.85 (2.47)	7.89 (2.64)
No fast fashion	3	0.12	4.67 (2.59)	8.22 (2.56)
Power strip	3	0.12	2.92 (1.95)	6.87 (3.09)
Print two-sided	3	0.12	3.03 (2.18)	7.20 (3.15)
Repair	3	0.12	5.24 (2.57)	7.70 (2.52)
2good2go (Foodsharing app)	2	0.08	3.87 (2.33)	6.92 (2.82)
Bamboo toothbrush	2	0.08	4.26 (2.55)	5.66 (3.01)
Bio garbage bag	2	0.08	2.84 (2.13)	7.06 (3.29)
Candle instead of electric light	2	0.08	6.99 (2.93)	5.06 (3.05)
No coffee to go (take your own)	2	0.08	3.86 (2.34)	7.07 (2.83)
Dishwasher (instead of washing by hand)	2	0.08	3.61 (2.70)	6.72 (3.21)
Fairteiler (Foodsharing facility)	2	0.08	4.58 (2.17)	7.02 (2.63)
Ice cream waffle (no cup)	2	0.08	2.51 (2.11)	6.65 (3.18)
Avoiding palm oil in products	2	0.08	4.17 (2.39)	7.16 (2.91)
No pizza service (get it yourself)	2	0.08	4.45 (2.52)	6.26 (2.87)
Ecological toilet paper	2	0.08	3.79 (2.25)	6.53 (3.13)
Reusable diaper	2	0.08	5.99 (2.49)	6.26 (3.15)
Forgo ordering food	2	0.04	4,51 (2.29)	8.02 (2.67)
Use bottle deposit	1	0.04	3.04 (2.12)	8.11 (2.99)
Cut vegetables yourself (no ready-to-cook food)	1	0.04	2.51 (1.87)	7.19 (3.23)
Do not throw gum on street	1	0.04	1.89 (1.72)	6.98 (3.25)
Recycle hazardous waste	1	0.04	4.89 (2.60)	7.40 (2.88)
Ignore best before date (bakery)	1	0.04	3.26 (2.30)	6.71 (3.00)
Music download (no stream)	1	0.04	4.45 (2.59)	5.85 (2.83)
No avocado (no exotic fruits)	1	0.04	4.76 (2.58)	6.61 (3.20)
No charging overnight (phone)	1	0.04	4.46 (2.77)	5.72 (3.05)
No softener	1	0.04	2.92 (2.04)	6.47 (3.12)
Do not use paper to wrap a present	1	0.04	4.16 (2.57)	5.30 (2.94)
Reusable period products	1	0.04	5.03 (3.03)	6.49 (3.22)
Reuse PET bottles	1	0.04	3.05 (2.23)	6.92 (3.08)
Save fuel	1	0.04	3.94 (2.26)	7.76 (2.87)
Skitour (no lifts)	1	0.04	5.79 (2.65)	6.57 (2.77)
Overall	2,507	100		

#### Participants

Between June 21 and August 9, 2021, 163 participants took part in Study 2, which was advertised in the University Newsletter and on Facebook. The study was carried out using the online software SoSci survey (Leiner, 2019). As a reward, the participants could receive course credit or take part in a raffle to win one of three cash prizes of 50 Euros, by finishing the study. The study was carried out online. Four participants had to be excluded from further analyses because at least one of two attention check items was answered incorrectly (resulting *N* = 159). The participants included 100 women and 57 men, as well as two participants with different gender. They were on average 24.75 (*SD* = 8.91) years old, between the ages of 18 and 75. More than 90% (*N* = 147) of the participants had at least a high school degree as their highest educational qualification. Most of them lived in Germany and Austria (*N* = 142).

### Results

Table 3 shows Spearman correlations and Cronbach’s alpha of the variables. Reliability of the light triad scale as a whole was *α* = 0.71 and *α* = 0.87 for the dark tetrad scale.

**Table 3:** Spearman’s rank rho correlations matrix as well as reliabilities (Cronbach’s alpha; diagonal) of Study 2 (*N* = 159).

		*M*	*SD*	1	2	3	4	5	6	7	8
1	PEA	4.58	0.61	0.79							
2	MACH	3.09	0.86	−0.19^*^	0.73						
3	NARC	3.14	0.8	−0.28^**^	0.34^**^	0.76					
4	SAD	2.06	0.83	−0.31^**^	0.41^**^	0.25^**^	0.78				
5	PSYC	2.34	0.79	−0.26^**^	0.53^**^	0.43^**^	0.60^**^	0.69			
6	HUMAN	4.74	0.72	0.30^**^	−0.18^*^	−0.14	−0.22^**^	−0.23^**^	0.58		
7	FIH	4.07	0.96	0.29^**^	−0.27^**^	0.08	−0.24^**^	−0.27^**^	0.41^**^	0.72	
8	KANT	4.28	0.89	0.14	−0.26^**^	−0.17^*^	−0.11	−0.21^**^	0.24^**^	0.24^**^	0.48

M, arithmetic mean; SD, standard deviation; PEA, pro-environmental attitude; MACH, Machiavellianism; NARC, narcissism; SAD, sadism; PSY, psychopathy; HUMAN, humanism; FIH, faith in humanity; and KANT = Kantianism.

* Level of significance: *p* < 0.05.

** Level of significance: *p* < 0.01.

In a first step, we repeated the regression analyses of Study 1 with the data from Study 2. Estimated behavioral costs and benefits served as a dependent variable, predicted by personality and attitude traits (one model for each predictor). The results of these linear regression models showed that pro-environmental attitude was negatively related to behavioral costs, *β* = −0.35 *CI*_95%_ [−0.50, −0.20], *p* < 0.001, and positively related to behavioral benefits, *β* = 0.28 *CI*_95%_ [0.13, 0.43], *p* = 0.001. The dark tetrad was positively associated with behavioral costs, *β* = 0.31 *CI*_95%_ [0.16, 0.46], *p* < 0.001, but not significantly related to behavioral benefits, *β* = −0.13 *CI*_95%_ [−0.29, 0.03], *p* = 0.119. Regression analyses for the light triad showed a positive relationship with behavioral benefits, *β* = 0.16 *CI*_95%_ [0.00, 0.32], *p* = 0.047, and a negative relationship with behavioral costs, *β* = −0.20 *CI*_95%_ [−0.35, −0.04], *p* = 0.012. In summary, similar to Study 1, less environmentally aware people were more likely to perceive PEB to be costly and of little benefit than pro-environmental people. Importantly, while Study 1 focused on how people rate their own behavior, Study 2 showed that the same pattern of data occurs when observers make the judgments.

In a next step, the cost and benefit estimates given by the participants of Study 2 were compared with the data from Study 1. With regard to behavior costs and benefits, the assessments of the actors and observers were positively correlated (costs: *r*_s_ = 0.21 [0.16, 0.25], *p* < 0.001; benefits: *r_s_* = 0.37 [0.32, 0.41], *p* < 0.001). On average, observers estimated a higher benefit (*M*_difference_ = 0.30, *SD* = 0.09) and cost (*M*_difference_ = 0.37, *SD* = 0.06) than actors. A Welch two sample t-test proved that these differences were significant (*M*_observers’ benefit_ = 5.63, *SD* = 1.95; *M*_actors’ benefit_ = 5.33, *SD* = 3.01; *p* = 0.001; *M*_observers’ cost_ = 1.93, SD = 1.61, *M*_actor’s cost_ = 1.56, *SD* = 1.90; *p* < 0.001).

Next, we examined the interaction of attitude and personality with type of estimation (actors’ assessment of Study 1 vs. the observers’ assessment of Study 2). There was no significant interaction between pro-environmental attitude and type of estimation regarding costs (*b* = 0.00 *CI*_95%_ [−0.02, 0.03], *p* = 0.712; model statistics: *F*(2822) = 0.27, *p* = 0.850). The cost assessments of the actors and observers were not significantly related to pro-environmental attitude (actors: *b* = 0.00 *CI*_95%_ [−0.01, 0.02], *p* = 0.870; observers: *b* = 0.01 *CI*_95%_ [−0.01, 0.02], *p* = 0.447; model statistics: *F*(1410) = 0.36, *p* = 0.696).

Regarding the benefits of PEB, there was a marginally significant interaction effect between pro-environmental attitude and type of estimation (*b* = −0.02 *CI*_95%_ [−0.03, 0.00], *p* = 0.061; model statistics: *F*(2819) = 3.13, *p* = 0.025). The pro-environmental attitude was positively associated with the benefit estimates of the actors (*b* = 0.02 *CI*_95%_ [0.01, 0.03], *p* = 0.001), but not significantly related to the observers’ benefit estimations (*b* = −0.01 *CI*_95%_ [−0.03, 0.00], *p* = 0.091; model statistics: *F*(1410) = 6.11, *p* = 0.002).

Examining the dark tetrad, a multiple linear regression model showed a significant interaction effect of cost estimations by actors and observers (*b* = −0.03, *CI*_95%_ [−0.06, −0.01], *p* = 0.005; model statistics: *F*(2886) = 8.45, *p* <0. 001). The actors’ perceived costs were positively related to the dark tetrad (*b* = 0.04 *CI*_95%_ [0.02, 0.05], *p* < 0.001), whereas the observers’ perceived costs were not significantly related to the dark tetrad scale (*b* = −0.01 *CI*_95%_ [−0.02, 0.01], *p* = 0.513; model statistics: *F*(1442) = 12.86, *p* < 0.001).

In line, benefit estimations interacted (*b* = 0.03, *CI*_95%_ [0.01, 0.04], *p* = 0.001; model statistics: *F*(2883) = 4.06, *p* = 0.002), as the actors’ benefit estimations were negatively related to the dark tetrad (*b* = −0.02 *CI*_95%_ [−0.03, −0.01], *p* < 0.001), but the observers’ benefit estimations were positively related (*b* = 0.02 *CI*_95%_ [0.01, 0.04], *p* = 0.002; model statistics: *F*(1442) = 11.31, *p* < 0.001).

Examining the light triad, there was an interaction effect of cost estimations (*b* = 03 *CI*_95%_ [0.01, 0.05], *p* = 0.002; model statistics: *F*(2904) = 12.5, *p* < 0.001), because actors’ perceived costs were negatively related to the light triad (*b* = −0.04 *CI*_95%_ [−0.05, −0.03], *p* < 0.001), but the observers’ perceived costs were not significantly related (*b* = 0.00 *CI*_95%_ [−0.01, 0.02], *p* = 0.638; model statistics: *F*(1451) = 18.44, *p* < 0.001).

**Figure 3:**
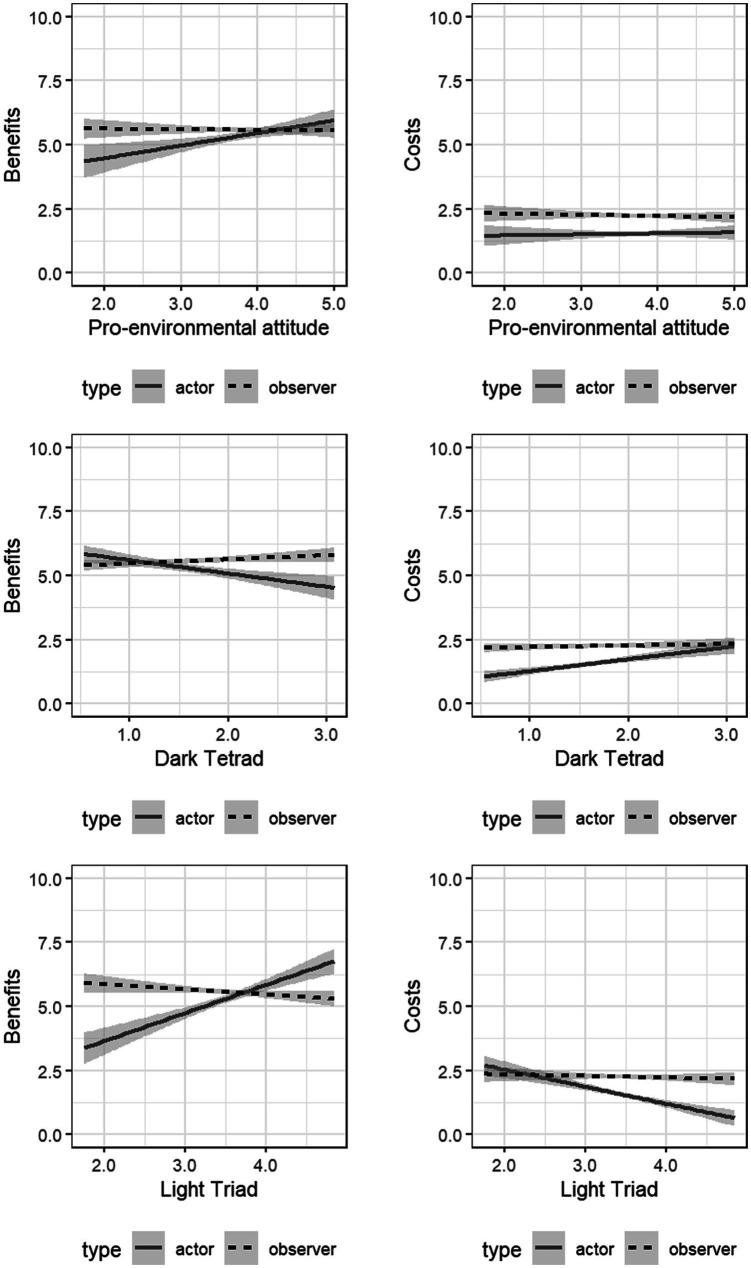
PEB’s benefit (left) and cost (right) estimates by participants of Study 1 (“actors,” black) and Study 2 (“observers,” grey dashed), dependent on pro-environmental attitude, the dark tetrad, and the light triad of personality. The grey-shaded area displays a *CI*_95%_.

Regarding the PEB’s benefits and the light triad, there was again an interaction effect of estimations (*b* = −0.03 *CI*_95%_ [−0.05, 0.02], *p* < 0.001; model statistics: *F*(2900) = 13.65, *p* < 0.001). The actors’ benefit estimations were positively related to the light triad (*b* = 0.03 *CI*_95%_ [0.02, 0.04], *p* < 0.001), but the observers’ benefit estimations were negatively related (*b* = −0.03 *CI*_95%_ [−0.04, −0.01], *p* < 0.001; model statistics: *F*(1451) = 29.58, *p* < 0.001). Figure 3 displays all outlined effects.

### Discussion

In Study 2, independent observers rated the PEBs of the actors in the first study. The results reflected the results of Study 1, since the attitudes and personality of the observers showed the same relations to cost and benefit estimates as the personality and attitudes of the actors themselves. Pro-environmental attitude and the light triad (of the observers) went along with increased estimates of PEB’s benefits and lower estimates of costs of the actors’ behaviors. In contrast, the observers’ dark personality was associated with increased estimates of behavioral costs, but was unrelated to the benefits of the actors’ behaviors. It turns out that not only actors (Study 1) differ in their perception of the costs and benefits of PEB depending on their pro-environmental attitude and personality, but also observers predominantly show the same pattern. Taken together, pro-environmental people find PEB to be of great benefits and low cost, regardless of whether the person shows the behavior or someone else does.

However, it was initially unclear whether costs and benefits are perceived differently or whether the actor’s PEB actually differs in how costly and beneficial the behavior was. For example, a person with a relatively high score in the dark tetrad may find their own PEB costly and not beneficial (but others would disagree) and/or the PEB was actually costly and not beneficial (i.e., others would agree). If the latter were to be true, the findings of Study 1 would suggest that dark people are indeed involved in more costly environmental decisions. To disentangle these two accounts, we combined Studies 1 and 2 and tested whether the actor’s perception of their PEB differed from the observer’s perception, depending on the actor’s pro-environmental attitude and their dark and light personalities.

Study 1 showed that participants with low levels on pro-environmental attitude and the light triad and high levels on the dark tetrad found their PEB to be relatively costly and unbeneficial. However, the results showed that observers (Study 2) did not share this view. Indeed, actors’ and observers’ estimates of the costs and benefits of PEB differed systematically: less environmentally aware actors in Study 1 generally estimated the costs of behavior to be higher than the independent observers. In contrast, pro-environmental actors estimated the behavioral benefits to be significantly higher, while the behavioral costs were estimated to be lower, compared to the observers’ perception. Overall, compared to independent observer ratings, pro-environmental people tend to underestimate the costs and overestimate the benefits of their PEB, whereas the opposite is true for less environmentally aware people.

## General Discussion

The present research examined the interplay between personality and contextual aspects of PEB. Our aim was to investigate how pro-environmental attitude and the dark and light sides of personality are related to PEB in daily life and the potential moderating role of the associated costs and benefits. We expected attitude and personality not to be predictive when PEB is mainly beneficial, but when costs are high and benefits are low, only pro-environmental people should engage in PEB.

Since the relationship between attitude and behavior is complex (Bechler et al., 2021) and the “attitude–behavior gap” (Kollmuss and Agyeman, 2002, p. 246) suggests that concern about environmental problems does not necessarily mean that the corresponding behavior goes hand in hand, we used the ecological momentary assessment approach to further investigate the relationship between attitude, personality, and “green” behavior. This method minimizes recall biases and increases the ecological validity. In line with our hypotheses, we found that pro-environmental attitude and the light triad were positively associated with PEB in daily life, whereas the dark tetrad was associated with less mentions of daily PEB.

Unexpectedly, Study 1 also showed that less environmentally aware participants more frequently reported PEB with high cost and low benefit. Pro-environmental participants, on the other hand, reported more “beneficial” PEBs. These results suggest that non-environmentally aware people are less influenced by rational choice considerations than pro-environmental people. It is important that our cost–benefit measurements were based on how participants perceived their PEB, rather than an objective assessment of the actual costs and benefits. Therefore, in a second study, we tested whether independent observers would agree with how costs and benefits are perceived by the actors. In this way, we were able to investigate whether less environmentally aware people perceive higher costs and lower benefits of PEB than pro-environmental people do, or whether the behavior of less environmentally aware people was actually more costly and less beneficial.

In this second study, we then examined how actors and observers differ in their perception of costs and benefits associated with PEB, depending on the actor’s pro-environmental attitude and personality. In fact, the results strongly suggest that two people may perceive the same environmental behavior in completely different ways. Whether this behavior perceived as costly or beneficial depends on personality and attitude: while pro-environmental actors stated the behavioral benefits as significantly higher and the behavioral costs as lower than the observers’ estimates suggest, the less environmentally aware actors estimated the behavioral costs generally higher than the independent observer did. Therefore, we conclude that personality, particularly its light and dark side, is associated with biased situational judgments of the costs and benefits of PEB.

With regard to the practical implications, it can be assumed that PEB is not only perceived as beneficial by pro-environmental people but can actually also be good for their selves. In previous research, “green” behavior has often been associated with subjective wellbeing, suggesting that environmentally friendly behavior is beneficial not only for the environment, but also for personal happiness (Brown and Kasser, 2005; Corral-Verdugo et al., 2011; Kaida and Kaida, 2016). Conversely, the question arises whether less environmentally aware people feel worse about environmentally behavior, because it seems to be a great burden for them. It is also conceivable that environmentally harmful behavior reduces the wellbeing of pro-environmental people. Answering these questions is an important starting point for future studies. Moreover, to encourage PEB in everyday life, financial costs and benefits may provide a more objective framework for promoting pro-environmental behavior than subjective behavioral costs, such as effort, effort, and time. Testing such an intervention can be a further task for future studies.

The present results also raise questions about the appropriateness of some popular methods used in scientific environmental psychology. What exactly counts as PEB can possibly be influenced by personality and attitude as well as associated cost and benefit estimates. Looking at the qualitative results of the present study (Table 2) it becomes clear that some behaviors may appear natural to some, while they represented a high hurdle for others. For example, the “lid (cooking)” category, that is, using a pot lid (instead of not doing it), was not worth mentioning for many participants, but others rated it as having high behavior costs (maximum: seven on a scale from 0 to 10). A person’s environmental awareness can therefore be rather subjective when using self-report questionnaires, even if certain behaviors are asked. Not only the environmental behavior itself, but also its stated frequency in validated questionnaires could be shaped by personality and attitude.

### Limitations and Future Research

The results are based on samples of European citizens mainly from Germany and Austria. Most of the participants were highly educated. Future research may examine the estimates of costs and benefits of PEB by participants with different ethnic backgrounds and with more diverse educational levels. These circumstances could have an impact on pro-environmental decision-making and estimates of associated costs and benefits. In addition, future research could aim to observe real-world behavior rather than repeated self-reports during the Ecological Momentary Assessment.

Additionally, people might not only misjudge the behavioral costs and benefits of specific PEBs, shaped by their personality and attitude, but also misjudge the actual environmental impact of these behaviors (Cologna et al., 2022). For example, it was shown that people underestimate the potential of switching to a sustainable diet, but overestimate the potential of recycling and avoiding plastic (e.g., Wynes et al., 2020). These expectations of environmental impact could also be biased by personality and attitude, as well as environmental knowledge. To investigate this hypothesis can be another topic for future research.

### Conclusion

In summary, the present results support the subjectivity of the costs and benefits associated with PEB. Pro-environmental people (with strong pro-environmental attitude and/or light personalities) not only behave more environmentally friendly in everyday life, but also perceive PEB as a great benefit and less burden. In contrast, people who are less environmentally aware (with weak pro-environmental attitudes and/or dark personalities) perceive PEB to be costly and behave less environmentally friendly. Going green appears exhausting for dark personalities but benefitting for the light ones.

## Data Availability Statement

The raw data supporting the conclusions of this article will be made available by the authors, without undue reservation.

## Ethics Statement

Ethical review and approval was not required for the study on human participants in accordance with the local legislation and institutional requirements. The patients/participants provided their written informed consent to participate in this study.

## Author Contributions

JK and TG: conceptualization and methodology. JK: formal analysis, data curation, and writing—original draft preparation. TG: writing—review and editing. All authors contributed to the article and approved the submitted version.

## Funding

This publication was funded by the University of Innsbruck.

## Conflict of Interest

The authors declare that the research was conducted in the absence of any commercial or financial relationships that could be construed as a potential conflict of interest.

## Publisher’s Note

All claims expressed in this article are solely those of the authors and do not necessarily represent those of their affiliated organizations, or those of the publisher, the editors and the reviewers. Any product that may be evaluated in this article, or claim that may be made by its manufacturer, is not guaranteed or endorsed by the publisher.

## Footnotes

^1^ https://aspredicted.org/hk4wr.pdf
